# Sex-Specific Functional Connectivity in the Reward Network Related to Distinct Gender Roles

**DOI:** 10.3389/fnhum.2020.593787

**Published:** 2021-01-11

**Authors:** Yin Du, Yinan Wang, Mengxia Yu, Xue Tian, Jia Liu

**Affiliations:** ^1^Faculty of Psychology, Beijing Normal University, Beijing, China; ^2^Department of Psychology, Tsinghua Laboratory of Brain and Intelligence, Tsinghua University, Beijing, China

**Keywords:** functional connectivity, reward network, gender roles, orbitofrontal cortex, frontal pole, nucleus accumbens, subcallosal cortex

## Abstract

Gender roles are anti-dichotomous and malleable social constructs that should theoretically be constructed independently from biological sex. However, it is unclear whether and how the factor of sex is related to neural mechanisms involved in social constructions of gender roles. Thus, the present study aimed to investigate sex specificity in gender role constructions and the corresponding underlying neural mechanisms. We measured gender role orientation using the Bem Sex-Role Inventory, used a voxel-based global brain connectivity method based on resting-state functional magnetic resonance imaging to characterize the within-network connectivity in the brain reward network, and analyzed how the integration of the reward network is related to gender role scores between sex groups. An omnibus analysis of voxel-wise global brain connectivity values within a two-level linear mixed model revealed that in female participants, femininity scores were positively associated with integration in the posterior orbitofrontal cortex and subcallosal cortex, whereas masculinity scores were positively associated with integration in the frontal pole. By contrast, in male participants, masculinity was negatively correlated with integration in the nucleus accumbens and subcallosal cortex. For the first time, the present study revealed the sex-specific neural mechanisms underlying distinct gender roles, which elucidates the process of gender construction from the perspective of the interaction between reward sensitivity and social reinforcement.

## Introduction

Gender roles are malleable and socially constructed phenomena (Keener and Mehta, 2017). Although androgynous individuals—those who possess both masculine and feminine characteristics (Lips, 2016)—may be more adaptive and flexible in multiple social contexts, evident in behaviors such as exhibiting emotional stability in volatile situations and effectively dealing with stress at work (Gartzia et al., 2018; Yu et al., 2020), the masculine male and feminine female may still be the most socially desirable (Auster and Ohm, 2000; Prentice and Carranza, 2002). The social constructions of femininity and masculinity are entangled with biological sex, which is implied in the definition of these characteristics (Bem, 1974). Specifically, feminine traits were described as significantly more desirable for a female than for a male individual, whereas masculine traits were categorized as significantly more desirable for a male than for a female individual (Auster and Ohm, 2000). From this perspective, although females and males should be encouraged to construct masculine and feminine characteristics independent of sex as a consideration, the actual social construction process and the social desirability of feminine and masculine gender roles are sex-dependent.

The traits deemed socially desirable seem to be deeply and culturally engrained (Auster and Ohm, 2000). In behavioral research, results have been inconsistent regarding whether gender constructs have changed as society has changed (Auster and Ohm, 2000; Prentice and Carranza, 2002; Seem and Clark, 2006; Dean and Tate, 2017). Whereas the results of some studies suggest that the desirability of particular traits for males and females may be changing along with other changes in society (Dean and Tate, 2017), the results of other studies seem to reflect the persistence of traditional gender role expectations for men and women (Auster and Ohm, 2000; Prentice and Carranza, 2002; Seem and Clark, 2006). Because of inconsistency in behavioral research regarding gender constructions, studies at the neural level may elucidate both the role of biological sex and sociocultural factors in the development of gender roles. For example, a recent study combining both resting-state functional connectivity and behavioral data (i.e., gender role orientation) obtained from cisgender (men/women) and transgender (trans men/trans women) individuals and using canonical correlation analyses captured nine brain–gender axes (i.e., canonical vectors) across the four participant groups (Clemens et al., 2020). Investigating the neurobiological underpinnings of gender types beyond the common gender dichotomization is informative but may not reveal the sex-specific social construction of gender characteristics and the corresponding neural mechanisms; thus, the current study aimed to explore the sex-specific neural signatures underlying gender constructions.

The construction of gender roles is a process in which individuals learn and internalize the social desirability of and preference for specific gender characteristics (Tang and Tang, 2001). The constant interactions between reward processing and social reinforcement contribute to the construction of masculinity and femininity (Ruble et al., 2007; Losin et al., 2012). Thus, an individual’s greater sensitivity to social reward and social approval makes him or her more effective at processing and internalizing the socially desirable norms of a specific gender role (Jones et al., 2014). This line of reasoning suggests that those individuals who are sensitive in reward processing tend to seek to present themselves in the most favorable light and then gradually come to possess socially valued characteristics (Dölen et al., 2013; Han and Ma, 2015; Schultz, 2015). Relevant research has provided intriguing evidence that reward sensitivity can promote social norm acquisition (Han and Ma, 2015; Kitayama and Huff, 2015). For example, people who carry a 7- or 2-repeat allele of the dopamine D4 receptor gene (*DRD4*) are more sensitive to reward than are those who do not carry this allele (Stice et al., 2010; Glimcher, 2011). Based on this specific feature of *DRD4*, recent work has suggested that the carriers of a 7- or 2-repeat allele are more likely to exhibit culturally typical response patterns compared with non-carriers (Kitayama et al., 2014, 2016), which means that social norm acquisition is promoted by reward processing sensitivity.

Reward processing consists of multiple aspects or stages, such as reward anticipation (e.g., incentive salience to rewards), learning the motivational value of rewards (e.g., conditioned rewards or extrinsic rewards), and reward consumption (e.g., hedonic reactions to rewards; Gottfried et al., 2003; Rademacher et al., 2010; Wang et al., 2016; Lichtenberg et al., 2017). All these aspects involved in reward processing induce a synergistic functional change in the brain reward system (Camara et al., 2009; Oldehinkel et al., 2016). To assess the sensitivity of reward processing, the use of resting-state functional magnetic resonance imaging (rs-fMRI) is a promising approach to measure the functional connectivity (FC) in a specific brain network [i.e., the reward network (RN); Damoiseaux et al., 2006; Van den Heuvel and Hulshoff Pol, 2010]. Several studies have indicated that high levels of FC exist in known functional networks, such as the face processing and language networks (Simmons and Martin, 2011; Hutchison et al., 2014; Stevens et al., 2015; Wang et al., 2016; Yu et al., 2017). More crucially, stronger correlations in a specific functional network are associated with superior performance in specific functional behavior tasks (Hutchison et al., 2014; Wang et al., 2016; Yu et al., 2017).

To investigate the sex-specific neural mechanisms that underlie distinct gender role constructs in the brain RN, we first characterized the FC of the RN in a large sample of participants (*N* = 272) by using a voxel-based global brain connectivity (GBC) method with rs-fMRI (Cole et al., 2012). Considering neuroimaging studies have revealed that multiple brain regions, including the ventral striatum [i.e., nucleus accumbens (NAc)], dorsal striatum (i.e., caudate and putamen), amygdala, orbitofrontal cortex (OFC), and subcallosal cortex (SCC), are coactivated in a variety of reward processing tasks (Kringelbach, 2005; Dreher et al., 2008; Sescousse et al., 2010, 2013; Ruff and Fehr, 2014; Schultz, 2015; Li et al., 2016; Wang et al., 2016), the RN was defined as a set of voxels selectively responsive to reward processing through an automated meta-analysis (Kong et al., 2017). Subsequently, the functional integration of the RN was determined by calculating the within-network connectivity (WNC) of each voxel in the RN. Specifically, the WNC was calculated as the averaged FC of a voxel to the remaining reward-selective voxels in the RN. By correlating the WNC of each voxel in the RN with the scores of masculinity and femininity across males and females within a two-level linear mixed model (LMM), we characterized the sex-specific gender role relevance of the integration (i.e., a stronger WNC) of the RN, thereby aiming to elucidate the sex specificity underlying social constructions and social preferences of gender roles.

## Materials and Methods

This study investigated the sex-group specificity between gender roles and RN integration, which is an effective indicator for measuring the processing sensitivity of specific brain functions (Hutchison et al., 2014; Stevens et al., 2015). First, we conducted an automated meta-analysis on the term “reward” to localize the RN (Yarkoni et al., 2011; Kong et al., 2017). Second, an rs-fMRI scan was used to characterize the intrinsic WNC of the RN. Third, we used the Bem Sex-Role Inventory (BSRI; Bem, 1974) outside of the MRI scanner in a separate behavioral session to measure the gender role orientation for each participant (using the same cohort as in the rs-fMRI scan). Finally, an omnibus analysis of WNC values within the two-level LMM was performed to investigate the sex-specific gender role relevance of the RN’s integration.

### Participants

In total, 272 participants [146 females; 272 self-reported right-handed; mean age = 20.4 years, standard deviation (SD) = 0.9 years] from Beijing Normal University in Beijing, China, participated in this study as part of an ongoing project investigating associations among genes, the environment, the brain, and behavior (Wang et al., 2016; Chen et al., 2017; Yu et al., 2018; Wu et al., 2020). Participants were instructed to undertake a series of computer-based cognitive ability tests, paper–pencil questionnaires, and MRI scans. The computer-based cognitive ability tests assessed respondents’ abilities mainly involved in reasoning, attention, memory, object/face recognition ability, spatial ability, musical ability, and language skills. The paper–pencil questionnaires mainly assessed their family environment (e.g., socioeconomic status), school environment (e.g., teaching styles), and personalities (e.g., the BSRI). Data that were not relevant to the theme of this study are not reported here. Participants completed the fMRI scan first, which was followed by behavioral tests within several weeks. The participants reported no history of neurological or psychiatric disorders and had a normal or corrected-to-normal vision. The Institutional Review Board of Beijing Normal University approved both the behavioral and MRI protocols. Written informed consent was obtained from all the participants before the study.

### Reward Network Map From Neurosynth Meta-Analysis

To obtain an activation map relevant for reward processing, we used an automated meta-analysis tool called Neurosynth^1^ (Yarkoni et al., 2011) to generate an association test map displaying brain regions preferentially related to the term “reward.” The meta-analysis was performed by automatically identifying all studies in the Neurosynth database that loaded highly on the term. Meta-analyses were then performed to identify brain regions consistently or preferentially reported in the tables of those studies. The association test map for reward displayed voxels reported more often in the neuroimaging literature that included the term “reward” in their abstracts than in articles that did not include the term in their abstract. Despite the automaticity and potentially high noise resulting from the association between the term frequency and coordinate tables, this approach has been demonstrated to be robust and reliable (Yarkoni et al., 2011; Kong et al., 2017). The database was accessed in May 2018, the feature “reward” was searched for (922 studies with 30,418 activations), and the generated reward map was corrected using a false discovery rate (FDR) approach with an expected FDR of 0.01. As expected, the resulting statistical map included the bilateral ventral striatum (i.e., NAc), dorsal striatum (i.e., caudate and putamen), amygdala, OFC, and SCC, which is similar to the results obtained in previous reward-processing studies (Kringelbach, 2005; Dreher et al., 2008; Sescousse et al., 2010, 2013; Ruff and Fehr, 2014; Schultz, 2015; Li et al., 2016; Wang et al., 2016).

### Image Acquisition

MRI scanning was conducted using a 3T scanner (MAGNETOM Trio, A Tim System; Siemens) with a 12-channel phased-array head coil at Beijing Normal University Imaging Center for Brain Research, Beijing, China. The rs-fMRI scanning was conducted using the gradient-echo echo-planar imaging sequence (repetition time = 2,000 ms, echo time = 30 ms, flip angle = 90°, number of slices = 33, voxel size = 3.125 × 3.125 × 3.6 mm^3^). Scanning lasted for 8 min and consisted of 240 contiguous echo-planar imaging volumes. During the scan, participants were instructed to relax without engaging in any specific task and to remain still with their eyes closed. Moreover, a high-resolution T1-weighted magnetization prepared gradient-echo sequence (repetition time/echo time/inversion time = 2,530/3.39/1,100 ms, flip angle = 7°, matrix = 256 × 256, number of slices = 128, voxel size = 1 × 1 × 1.33 mm^3^) anatomical scan was acquired for registration purposes and anatomically localizing the functional regions. Earplugs were used to attenuate scanner noise, and a foam pillow and extendable padded head clamps were used to restrain participants’ head motion.

### Image Preprocessing

The rs-fMRI data were preprocessed using the Functional Magnetic Resonance Imaging of the Brain Software Library (FSL^2^). The preprocessing included the removal of the first four images, head motion correction (by aligning each volume to the middle volume of the image with the MCFLIRT), spatial Gaussian smoothing (with a Gaussian kernel of 6 mm full-width at half-maximum), intensity normalization, and the removal of linear trends. A temporal bandpass filter (0.01–0.1 Hz) was then applied to reduce low-frequency drifts and high-frequency noise.

To further eliminate physiological noise, such as the fluctuations caused by motion, cardiac and respiratory cycles, nuisance signals from cerebrospinal fluid, white matter, whole-brain average, motion correction parameters, and the first derivatives of these signals were regressed out using the methods described by Fox et al. (2005) and Biswal et al. (2010). The four-dimensional residual time series obtained after removing the nuisance covariates was used for the rs-FC analyses. The strength of the intrinsic FC between two voxels was estimated using the Pearson’s correlation of the residual resting-state time series for those voxels.

The registration of each participant’s rs-fMRI images to the structural images was conducted using FLIRT to produce a 6 degrees-of-freedom affine transformation matrix. The registration of each participant’s structural images to a common stereotaxic space [the Montreal Neurological Institute (MNI) 152-brain template with a resolution of 2 × 2 × 2 mm^3^, MNI152] was accomplished using FLIRT to produce a 12-degrees-of-freedom linear affine matrix (Jenkinson and Smith, 2001; Jenkinson et al., 2002).

### Behavioral Tests

#### Gender Role Orientation

The gender role orientation was measured using the modified Chinese version of the BSRI (Bem, 1974; Zhang et al., 2001). Of the 16 items in the short form BSRI, eight items relate to a masculine orientation (e.g., independent, assertive, and forceful), and eight relate to a feminine orientation (e.g., understanding, sympathetic, and compassionate). Respondents were asked to evaluate themselves on a six-point Likert scale ranging from 1 (never or almost never true) to 6 (always or almost always true). In the current study, the Cronbach’s alpha of the masculinity subscale was 0.826, and that of the femininity subscale was 0.797. Androgyny was calculated by subtracting the difference between the masculine and feminine scores from their sum (M + F − |M − F|) (Donnelly and Twenge, 2016).

### Within-Network Connectivity Analyses in the Reward Network

#### Within-Network Connectivity Estimation

The GBC method, which is a recently developed analytical approach for neuroimaging data, was used to characterize the intrinsic WNC of each voxel within the RN (Cole et al., 2012). The GBC of a voxel was generally defined as the averaged FC of that voxel to the remaining voxels in the entire brain or a predefined mask. This method enabled the characterization of a specific region’s full-range FC with voxel-wise resolution. In the present study, the WNC of each voxel in the RN was computed as the average FC of that voxel with the rest of the voxels within the RN. Then, participant-level WNC maps were transformed to *z*-score maps by using Fisher’s *z*-transformation to yield normally distributed values (Cole et al., 2012; Gotts et al., 2013). A one-sample *t*-test was performed for each voxel WNC to investigate the hub areas in the RN for both sex groups. The well-established regions identified in previous studies to be involved in reward processing were identified as regions of interest (ROIs), including the bilateral NAc, caudate, putamen, amygdala, OFC, and SCC (based on a 25% probability mask from the Harvard–Oxford cortical and subcortical probability atlas provided in FSL). If the WNC value of an area was 1 SD higher than the mean WNC value of the RN, this suggested that it is a hub of the RN (Dai et al., 2014). Moreover, we conducted two-sample *t*-tests to compare the WNC in the RN between male and female participants for testing whether differences existed in the RN integration between sexes. Significance was determined using FDR correction with *p* < 0.05.

### Within-Network Connectivity–Gender Role Correlation Analyses

An omnibus analysis within the two-level LMM was conducted to examine the sex specificity between the WNC of each voxel in the RN and the individual differences in gender role orientation. Specifically, a random slope–random intercept model was used, with WNC in the RN set as the dependent variable and gender role scores (i.e., femininity and masculinity) set as predictors on the first level and sex set as a predictor on the second level. A cross-level interaction occurred when the random slope of a Level 1 predictor was predicted by a Level 2 predictor (Preacher et al., 2006). The model has been implemented with Functional Magnetic Resonance Imaging of the Brain’s Local Analysis of Mixed Effects Stage 1 in FSL (Beckmann et al., 2003; Woolrich et al., 2004, 2009) with four contrasts for testing the cross-level interaction hypothesis. The inference of interest was whether the linear relationship between the dependent variable and gender role scores differed between sexes. In particular, to compare the difference in linear relationships between the dependent variable and feminine scores in females (Slope 1) and males (Slope 2) as well as the difference in the linear relationships between the dependent variable and masculine scores in females (Slope 3) and in males (Slope 4), four contrasts were set (i.e., Slope 1 > Slope 2, Slope 2 > Slope 1, Slope 3 > Slope 4, Slope 4 > Slope 3). Then, exploratory analyses were conducted to probe the interaction further, which provided simple slopes of the dependent variable (i.e., WNC) regressed on Level 1 predictors (i.e., feminine and masculine scores) at each conditional value of Level 2 predictors (i.e., female and male separately). Multiple-comparison correction was performed on the statistical map using 3dClustSim implemented in Analysis of Functional NeuroImages^3^. A threshold of cluster-level *p* < 0.05 and voxel-level *p* < 0.01 was set based on Monte Carlo simulations in the RN mask. Because cluster-extent-based thresholding might provide low spatial specificity (i.e., it might produce large clusters spanning multiple anatomical regions; Woo et al., 2014), to verify the spatial precision of the brain regions associated with distinct gender roles in different sex groups, we overlapped the corrected cluster with predefined ROIs to identify the cluster that mainly included which regions in the RN.

Furthermore, to probe the specificity of gender role effects for the RN, we calculated the WNC in the dorsal attention network (DAN) and motor network (MN) (as control networks) (Supplementary Figure 1A). Using the same protocol as we used to generate an RN map via Neurosynth, the feature of “dorsal attention” was searched for (99 studies with 3,720 activations), and that of “motor network” was searched for (85 studies with 3,811 activations); the generated DAN and MN maps were corrected using the FDR approach with an expected FDR of 0.01. Then, mixed model analyses were performed in the DAN (and MN) within the two-level LMM using the same parameter settings and correction method as for the analysis conducted in the RN.

Finally, control analyses were performed to rule out the possible confounding factor (i.e., head motion) in all analyses. Because relevant studies have indicated that rs-FC is strongly affected by head motion (Power et al., 2012; Van Dijk et al., 2012), we calculated a partial correlation between WNC and gender role scores while controlling for head motion. The extent of head motion was measured using the mean framewise displacement for each participant (Van Dijk et al., 2012).

### Participant Exclusion

Quality control of the MRI data focused on the artifacts caused by head motion during scanning. Participants whose head motion is > 3° in rotation or 3 mm in translation throughout the fMRI scan should be excluded from further analyses. For the rs-fMRI, no participants were excluded according to this criterion.

## Results

### Behavior Results

**FIGURE 1:**
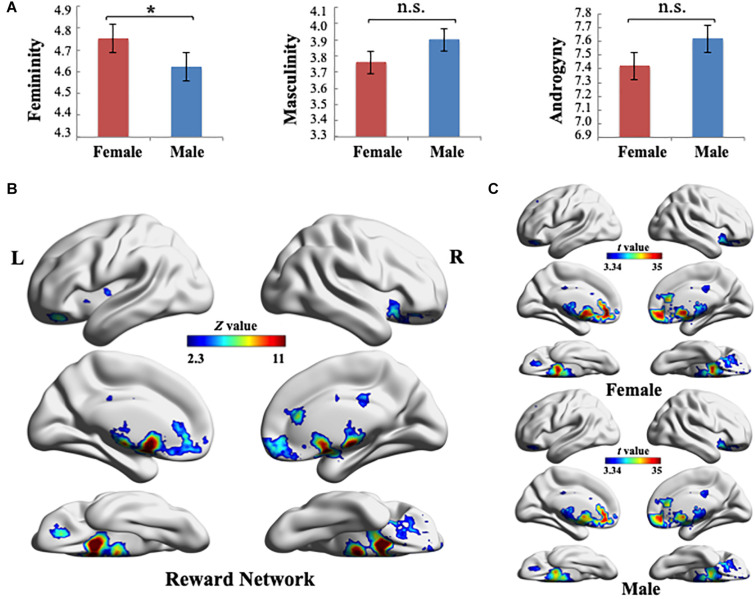
**(A)** Gender role scores were measured using the Bem Sex-Role Inventory. One-way analysis of variance results indicated that significant differences exist in the femininity between sex groups (*p < 0.05). Error bars indicate ± 1 standard mean error. **(B)** Reward-processing map created in Neurosynth meta-analysis (Z > 2.3, uncorrected). Ten reward-selective regions of interest were included in this reward network mask, including the ventral striatum (i.e., bilateral nucleus accumbens), dorsal striatum (i.e., bilateral caudate and putamen), bilateral amygdala, orbitofrontal cortex, and subcallosal cortex. **(C)** Global pattern of the within-network connectivity (WNC) in the reward network. Group-level (one-sample *t*-test) WNC map in females (top) and group-level (one-sample *t*-test) WNC map in males (bottom) are overlaid on the cortical surface (*t* > 3.34, two-tailed *p* < 0.001, uncorrected). L, left; R, right. Visualization created using BrainNet Viewer (http://www.nitrc.org/projects/bnv/).

The participants’ gender role scores were measured using the BSRI. Table 1 summarizes the descriptive statistics for the test. First, Pearson’s correlations were run to examine the relationship of masculinity (M) with femininity (F) for each sex group. No significant correlation was found to exist between F and M in either of the groups (female: *r* = 0.074, *p* = 0.377; male: *r* = −0.054, *p* = 0.547), which was consistent with Bem’s theory that M and F were two independent scales. Second, a one-way analysis of variance was used to examine potential group differences in F, M, and androgyny (M + F − |M − F|) between females and males. The results indicated that significant differences existed in F between sexes [*F*(1, 271) = 4.415, *p* = 0.037]. Participants demonstrated similar gender role orientations for M [*F*(1, 271) = 3.21, *p* = 0.074] and androgyny [*F*(1, 271) = 2.041, *p* = 0.157] (Figure 1A).

**TABLE 1:** Descriptive statistics of the behavior test for the two sex groups.

	Femininity		Masculinity		Androgyny	
Sex	Mean	*SD*	Mean	*SD*	Mean	*SD*
Female (*N* = 146)	4.75*	0.55	3.76	0.69	7.42	1.28
Male (*N* = 126)	4.62*	0.49	3.90	0.62	7.62	1.04

Means in the same column with * differed significantly at p < 0.05 in the analysis of variance.

### Definition of the Reward Network

To define the RN, we used the results of the Neurosynth meta-analysis and created an RN mask (*Z* > 2.3, uncorrected) according to the association test map. The RN mainly included the bilateral NAc, caudate, putamen, amygdala, OFC, and SCC (Figure 1B). The regions in the RN were in agreement with the reward-selective regions identified in studies on reward processing (Kringelbach, 2005; Dreher et al., 2008; Sescousse et al., 2010, 2013; Ruff and Fehr, 2014; Schultz, 2015; Li et al., 2016; Wang et al., 2016).

### Within-Network Connectivity in the Reward Network

After identifying the RN, we computed each voxel’s WNC in the RN using the rs-fMRI data, where the WNC measured the voxel-wise FC within the RN. First, we used a one-sample *t*-test to calculate the WNC across voxels in the entire sample (*N* = 272) and overlapped the RN with the ROIs, including the bilateral NAc, caudate, putamen, amygdala, OFC, and SCC (based on a 25% probability mask from the Harvard–Oxford cortical and subcortical probability atlas provided in FSL), for identifying the anatomical coordinates of the peak *t*-value in all the ROIs (Table 2).

**TABLE 2:** Anatomical coordinates of the peak *t*-value of within-network connectivity in regions of interest within the reward network.

		Peak MNI coordinates			
ROIs	Voxels	*x*	*y*	*z*	Peak t
Left NAc	89	−8	8	−8	56.36
Right NAc	84	14	10	−8	57.38
SCC	467	8	26	−10	46.34
Left putamen	367	−14	8	−10	52.12
Right putamen	333	16	8	−10	51.27
Left caudate	372	−10	14	−4	53.41
Right caudate	387	14	14	−4	55.37
Left amygdala	86	−16	−2	−16	37.18
Right amygdala	202	16	−2	−16	34.50
OFC	664	26	10	−16	35.27

*WNC, within-network connectivity; ROIs, regions of interest; RN, reward network; NAc, nucleus accumbens; SCC, subcallosal cortex; OFC, orbitofrontal cortex.*

As displayed in Figure 1C, quantitative analysis (a one-sample *t*-test on females and males, separately) indicated that among all of the RN regions, NAc, putamen, caudate, and SCC had the largest WNC values in both sex groups (*t* > 3.34, two-tailed *p* < 0.001, uncorrected). Furthermore, the WNC value of the bilateral NAc was one SD higher than the mean WNC value of the RN, which suggested that these regions are hubs of the RN (both in females and males) (Dai et al., 2014). In addition, a two-sample *t-*test between males and females across voxels in the WNC value within the RN revealed no significant differences between the sexes, which indicated that the integration of the RN was similar between males and females.

### Correlation Between Gender Roles and Within-Network Connectivity

After characterizing the WNC of the RN, we examined whether and how the integration of the RN was related to gender role scores between sex groups by conducting an omnibus analysis of voxel-wise WNC values in the RN within the two-level LMM.

**FIGURE 2:**
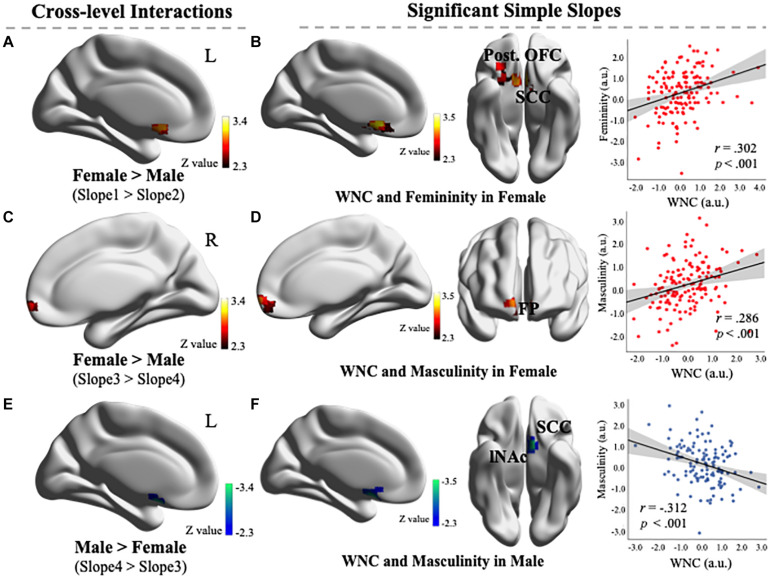
**(A)** Comparison of differences in the linear relationships between the within-network connectivity (WNC) values and feminine scores in females (Slope 1) and males (Slope 2) revealed that a cluster exhibited significant cross-level interaction in the contrast of Slope 1 > Slope 2 (54 voxels, Montreal Neurological Institute (MNI) coordinates: −8, 20, and −10). **(B)** A simple slope of regression between the WNC and femininity in females. One cluster’s WNC (located in the SCC and posterior OFC) was significantly positively correlated with femininity in females (295 voxels, voxel-level *p* < 0.01, corrected for multiple comparisons). Scatter plot depicts the correlations between the mean WNC in the entire cluster and the femininity (controlling for head motion). **(C)** A comparison of the difference in the linear relationships between the WNC values and masculine scores in females (Slope 3) and males (Slope 4) revealed that a cluster exhibited significant cross-level interaction in the contrast of Slope 3 > Slope 4 (75 voxels, MNI coordinates: −2, 62, and −8). **(D)** A simple slope of regression between the WNC and masculinity in females. One cluster’s WNC (located in the FP) was significantly positively correlated with masculinity in females (120 voxels, voxel-level *p* < 0.01, corrected for multiple comparisons). Scatter plot depicts the correlations between the mean WNC in the entire cluster and masculinity (controlling for head motion). **(E)** Comparison of differences in the linear relationships between the WNC values and masculine scores in males (Slope 4) and females (Slope 3) revealed that a cluster exhibited significant cross-level interaction in the contrast of Slope 4 > Slope 3 (45 voxels, MNI coordinates: −8, 10, and −16). **(F)** A simple slope of regression between the WNC and masculinity in males. One cluster’s WNC (located in the SCC and lNAc) was significantly negatively correlated with masculinity in males (81 voxels, voxel-level *p* < 0.01, corrected for multiple comparisons). Scatter plot depicts the correlations between the mean WNC in the entire cluster and masculinity (controlling for head motion). OFC, Orbitofrontal cortex; SCC, subcallosal cortex; FP, frontal pole; NAc, nucleus accumbens. L, left; R, right. a.u., arbitrary units. Visualization created using BrainNet Viewer (http://www.nitrc.org/projects/bnv/).

As illustrated in Figure 2A, a cluster exhibited significant cross-level interaction in the contrast of Slope 1 > Slope 2 (54 voxels, MNI coordinates of peak voxel: −8, 20, and −10 located in the SCC), which indicated that the slope between the voxel-wise WNC in this cluster and femininity for the female group was larger than that of the male group. Further exploratory probing of the cross-level interaction revealed that the simple slope of the WNC regressed on femininity was only significant in the female group. Specifically, a cluster in the SCC and posterior OFC (the spatial precision was verified by overlapping the cluster with ROIs) exhibited a significantly positive WNC–femininity correlation (Figure 2B; 295 voxels, partial *r* = 0.302, *p* < 0.001, MNI coordinates: −8, 20, and −10) in females. This exploratory result indicated that the female individuals with a stronger WNC (i.e., integration) in posterior OFC and SCC exhibited higher femininity. No cluster in the RN exhibited significance in the Slope 2 > Slope 1 contrast.

As illustrated in Figure 2C, a cluster exhibited significant cross-level interaction in the contrast of Slope 3 > Slope 4 [75 voxels, MNI coordinates of peak voxel: 2, 62, and −8 located in the frontal pole (FP)], which indicated that the slope between the voxel-wise WNC in this cluster and masculinity for the female group was larger than that of the male group. Further exploratory probing of the cross-level interaction revealed that the simple slope of the WNC regressed on masculinity was positively significant only in the female group. Specifically, a cluster in the FP (the spatial precision was verified by overlapping the cluster with a 25% probability mask from the Harvard–Oxford cortical probability atlas provided in the FSL) exhibited a significant positive WNC–masculinity correlation (Figure 2D; 120 voxels, partial *r* = 0.286, *p* < 0.001, MNI coordinates: −2, 62, and −8) in females. This exploratory result indicated that the female individuals with a stronger WNC (i.e., integration) in FP exhibited higher masculinity.

As illustrated in Figure 2E, a cluster exhibited significant cross-level interaction in the contrast of Slope 4 > Slope 3 (45 voxels, MNI coordinates of peak voxel: −8, 10, and −16 located in the SCC), which indicated that the slope between the voxel-wise WNC in this cluster and masculinity for the male group was larger than that of the female group. Further exploratory probing of the cross-level interaction revealed that the simple slope of the WNC regressed on masculinity was negatively significant only in the male group. Specifically, a cluster in the SCC and left NAc (the spatial precision was verified by overlapping with ROIs) exhibited a significant negative WNC–masculinity correlation (Figure 2F; 81 voxels, partial *r* = −0.312, *p <* 0.001, MNI coordinates: −8, 10, and −16) in males. This exploratory result indicated that male individuals with a stronger WNC (i.e., integration) in their NAc and SCC exhibited lower masculinity.

In addition, the control analysis conducted in the MN indicated that a cluster exhibited significance in the contrast of Slope 2 > Slope 1 (Supplementary Figure 1B; 193 voxels, MNI coordinates of the peak voxel: −26, −25, and 52 located in the precentral/postcentral gyrus). Exploratory probing of the cross-level interaction revealed that a cluster in the precentral/postcentral gyrus exhibited a significant positive WNC–femininity correlation (Supplementary Figure 1C; 274 voxels, partial *r* = 0.290, *p* = 0.001, MNI coordinates: −26, −25, and 52) only in males, which indicated that the male individuals with a stronger WNC (i.e., integration) in this cluster exhibited higher femininity. No significant correlation between the WNC in the DAN and gender role scores in both sex groups was evident after multiple-comparison correction.

## Discussion

In this study, we assessed the WNC in the RN in male and female groups and then investigated the sex-specific correlations between the WNC and gender role orientations. The results revealed that the WNC in the RN did not significantly differ between the sexes, which suggested that the functional integration within the RN was similar between male and female individuals. However, the mixed model regression analysis of the WNC and gender role scores demonstrated that female individuals with a stronger WNC in the posterior OFC and SCC (i.e., high integration) exhibited considerable feminine gender role orientation, whereas female individuals with a stronger WNC in the FP (i.e., high integration) exhibited a considerably masculine gender role orientation. By contrast, male individuals with a stronger WNC in the NAc and SCC exhibited lower masculinity. For the first time, the current study revealed that sex-specific neural mechanisms exist in the RN underlying distinct gender role constructs.

First, the finding of a stronger WNC of the NAc and SCC in the RN is consistent with previous findings that these brain regions play a central role in reward processing (Dreher et al., 2008; Sescousse et al., 2013; Ruff and Fehr, 2014; Schultz, 2015; Wang et al., 2016) and that the hub areas are similar in females and males, which implies that spontaneous integration of the RN is similar between sexes. However, our results revealed that sex-specific functional integration patterns in the RN were associated with distinct gender roles and indicated that different regions in the RN might be involved in the gender role constructions of each sex, suggesting that males and females’ distinct gender role characteristics might be reinforced by different types of rewards from their social environments (O’Doherty et al., 2001; Rademacher et al., 2010; Sescousse et al., 2010, 2013).

For the female group, the higher integration in the posterior OFC was associated with higher femininity, whereas the integration in the FP was positively correlated with masculinity. The OFC had relatively complicated functions in reward processing (O’Doherty et al., 2001; Howard et al., 2015), which means that the OFC was involved in distinguishing specific values of reward representations, with more complex or abstract reinforcers (e.g., monetary gains and social status) being represented more anteriorly in the OFC compared with less complex reinforcers (e.g., basic erotic stimuli; Kringelbach, 2005; Sescousse et al., 2013). The dissociation between OFC representations of primary and secondary rewards suggests an increasing trend in complexity along a posteroanterior axis according to abstract representations (Sescousse et al., 2010). In our exploratory results, integration in the posterior OFC was associated with females’ femininity, which indicated that the type of reward fed back to females who adhere to the social expectations of feminine gender roles might be limited to primary rewards (Sescousse et al., 2010, 2013). By contrast, integration in the FP was associated with masculinity in females, suggesting that the abstract positive reinforcement process was related to females’ masculinity (Yankouskaya et al., 2017), as the role of FP was involved in monitoring or evaluating self-generated decisions (Tsujimoto et al., 2009), maintaining abstract cognitive representations and action plans (Orr et al., 2019), and persisting with goal-directed behaviors (Hosoda et al., 2020).

For the male group, those with higher integration in the NAc demonstrated lower masculinity. The NAc, one part of the reward system, plays a crucial role in encoding the subjective value of rewards regardless of their type (Rademacher et al., 2010; Sescousse et al., 2014). The NAc is involved in the processing stages of reward anticipation and reward consumption (Wang et al., 2016). The functions of the NAc in reward processing suggest that men with high sensitivity in reward anticipation and reward consumption exhibit weakened masculinity irrespective of the reward type. Thus, society might render multiple types of rewards to men when they are inclined to decrease their masculinity. Because femininity and masculinity can be interpreted as interpersonal sensitivity and interpersonal potency, which refer to characteristics such as the abilities to accurately judge others’ behaviors and act as a leader (Brems and Johnson, 1990; Lambert and Hopwood, 2016), the latter personality trait (i.e., interpersonal potency) might be less beneficial in some social and work circumstances. Moreover, for males, endorsing more traditional masculine values may be associated with great costs in mental health (Mankowski and Maton, 2010).

In addition, the SCC was the only brain region related to gender roles in both sex groups. This observation is reasonable considering its specific function in reward processing (Elliott et al., 2000; Hebscher et al., 2015). The subcallosal cortical regions are thought to be involved in an early and automatic “feeling of rightness” in reward-based choice behavior (Elliott et al., 2000), which indicates that this region is involved in monitoring and “holding in mind” specific reward values. Therefore, the SCC is crucial for reward processing in social learning behavior and may represent a common neural mechanism underlying distinct gender role constructions in both sexes.

In conclusion, investigating sex-specific correlations between the WNC in the RN and gender role orientation is a starting point for exploring the ongoing process of complex and abstract social construction. The behavior results alone did not provide an indication of whether the similarity in masculinity between the sexes, which seems to exist, is caused by the social environment encouraging males to decrease their masculinity or because men today are less willing to endorse traits clearly associated with one gender vs. another (Donnelly and Twenge, 2016). Although, theoretically, gender roles should be constructed independently from biological sex, the present study’s investigation of sex-specific neural mechanisms underlying gender role characteristics elucidates the social desirability of and preference for femininity and masculinity by the sexes during gender construction in real social environments. Our results suggested that femininity and masculinity might be reinforced differentially between the biological sexes, and moreover, the role of sociocultural factors in the development of gender roles could manifest when individuals have higher functional integration in the reward processing network. Furthermore, from the perspective of brain plasticity (Lindenberger and Lövdén, 2019), our results may provide a possible explanation for the existent brain-based gender continuum (Clemens et al., 2020), which means that during the gender construction process, the brain is shaped both structurally and functionally in response to environmental feedback (Sale et al., 2014). Additionally, individuals’ biological sex, gender identity, and reward processing ability (e.g., the integration of the RN) are all factors involved in the constant brain–environment–behavior interactions, contributing to individuals’ brain-based gender variability.

### Limitations and Future Directions

Due to the cross-sectional nature of the present study, we were unable to depict the development of the neural basis for distinct gender roles between sexes. Some studies have suggested that sociotypical behavioral characteristics emerge after the age of approximately 6 years and become more pronounced throughout adolescence (Greenfield et al., 2003). Therefore, future longitudinal studies on children and adolescence would offer a deeper understanding of the developmental neural mechanisms of gender role characteristics. Furthermore, our study used a sample from China, which might limit the cross-cultural generalizability of its findings (Cyr and Head, 2013). Therefore, future cross-cultural research is required to examine whether the current findings can be generalized to Western samples.

## Conclusion

Our study provides the first empirical evidence demonstrating distinct gender roles related to sex-specific FC patterns in the RN. The exploratory results indicated that in females, femininity was associated with integration in the posterior OFC and SCC, whereas masculinity was correlated with integration in the FP. By contrast, masculinity was correlated with integration in the NAc and SCC in males. The current findings can help to delineate the sex-specific construction process of gender roles from the perspective of the interaction between social feedback and reward processing.

## Data Availability Statement

The raw data supporting the conclusions of this article will be made available by the authors, without undue reservation, to any qualified researcher.

## Ethics Statement

The behavioral and MRI protocols were approved by the Institutional Review Board of Beijing Normal University. The patients/participants provided their written informed consent to participate in this study.

## Author Contributions

YD contributed to the study conception and design, performed data analysis and interpretation, and wrote the manuscript. YW contributed to the study conception and design and critically revised the article. MY and XT performed the material preparation and the data collection. JL designed the work and critically revised the article. All authors contributed to the article and approved the submitted version.

## Conflict of Interest

The authors declare that the research was conducted in the absence of any commercial or financial relationships that could be construed as a potential conflict of interest.

## Footnotes

1 https://github.com/neurosynth/

2 http://fsl.fmrib.ox.ac.uk/fsl/fslwiki/

3 http://afni.nimh.nih.gov

## Supplementary Material

Supplementary Figure 1 **(A)** Dorsal attention network and motor network (MN) maps created in the Neurosynth meta-analysis (Z > 2.3, uncorrected). **(B)** Comparison of differences in the linear relationships between the within-network connectivity (WNC) values in the MN and feminine scores in males (Slope 2) and females (Slope 1) revealed that a cluster exhibited significance in the contrast of Slope 2 > Slope 1 (193 voxels, MNI coordinates: −26, −25, 52). **(C)** A simple slope of regression between the WNC and femininity in males. One cluster’s WNC (located in the precentral/postcentral gyrus) was significantly positively correlated with femininity in males (274 voxels, voxel-level *p* < 0.01, corrected for multiple comparisons). The scatter plot depicts the correlations between the mean WNC in the entire cluster and femininity (controlling for head motion). L, left; R, right. Visualization created using BrainNet Viewer (http://www.nitrc.org/projects/bnv/).
