# Supplementary figures and images for: Sex-Specific Functional Connectivity in the Reward Network Related to Distinct Gender Roles
Source: Front Hum Neurosci. 2021 Jan 11;14:593787. doi: 10.3389/fnhum.2020.593787 (PMC7831777; doi:10.3389/fnhum.2020.593787)

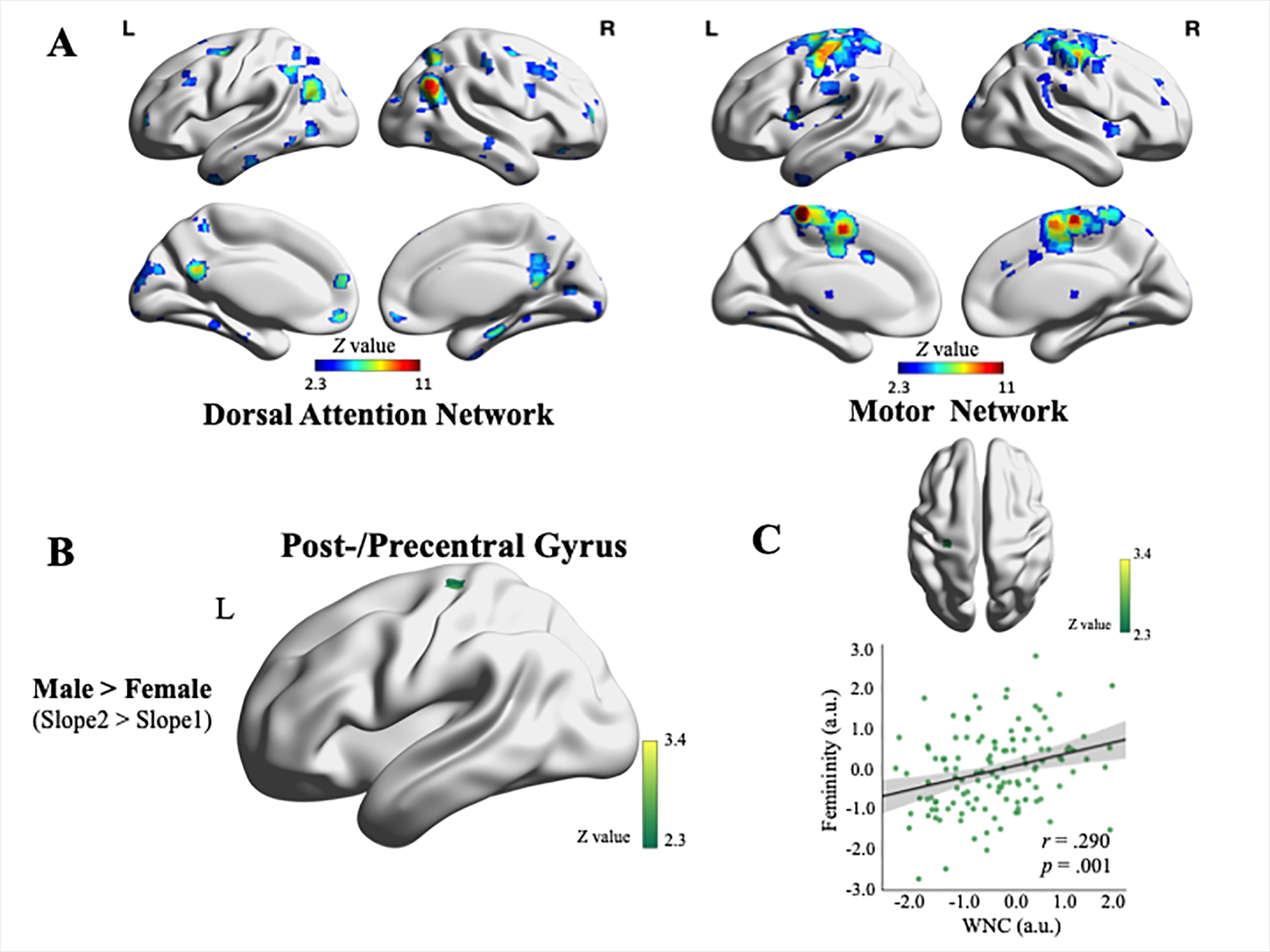

Supplement: Supplementary Figure 1 — (A) Dorsal attention network and motor network (MN) maps created in the Neurosynth meta-analysis (Z > 2.3, uncorrected). (B) Comparison of differences in the linear relationships between the within-network connectivity (WNC) values in the MN and feminine scores in males (Slope 2) and females (Slope 1) revealed that a cluster exhibited significance in the contrast of Slope 2 > Slope 1 (193 voxels, MNI coordinates: −26, −25, 52). (C) A simple slope of regression between the WNC and femininity in males. One cluster’s WNC (located in the precentral/postcentral gyrus) was significantly positively correlated with femininity in males (274 voxels, voxel-level p < 0.01, corrected for multiple comparisons). The scatter plot depicts the correlations between the mean WNC in the entire cluster and femininity (controlling for head motion). L, left; R, right. Visualization created using BrainNet Viewer (http://www.nitrc.org/projects/bnv/). [file Image_1.TIF]
